# Evaluation of the psychometric properties of the Italian version of the 10-item perceived stress scale in a sample of teachers

**DOI:** 10.3389/fpsyg.2023.1330789

**Published:** 2024-01-08

**Authors:** Linda Messineo, Crispino Tosto

**Affiliations:** Istituto per le Tecnologie Didattiche, Consiglio Nazionale delle Ricerche, Palermo, Italy

**Keywords:** COVID-19, perceived stress scale, positive affect and negative affect schedule, psychometric properties, self-reported health, teachers

## Abstract

**Background:**

The 10-item Perceived Stress Scale is a widely used questionnaire for measuring perceived stress.

**Aim:**

This study aimed to assess the psychometric properties of the Italian version of the 10-item Perceived Stress Scale in a sample of Italian teachers during the COVID-19 pandemic.

**Methods:**

A cross-sectional study was performed. A sample of 1,179 teachers of pre-primary, primary, and secondary schools anonymously completed an online questionnaire. A Confirmatory Factor Analysis was performed to compare the fit of a two-factor model against a one-factor solution of the scale. Multigroup CFA was run to test the measurement invariance of the two-factor PSS-10 solution across gender. The internal reliability of the scale was evaluated using Cronbach’s alpha and McDonald’ omega coefficients. Convergent validity with measures of subjective well-being and self-reported health was evaluated.

**Results:**

The results of the Confirmatory Factor Analysis showed that the two-factor structure of the 10-items Perceived Stress Scale provided a better fit to the data and supported the adequacy of the Italian version of the scale. The two-factor model showed measurement invariance across female and male groups, as result of the multigroup CFA. The scale proved to have good internal reliability. Correlation analyses with measures of the Positive Affect and Negative Affect Schedule and self-reported health supported convergent validity.

**Conclusion:**

These results suggest that the Italian version of the 10-item Perceived Stress Scale has good psychometric properties and can be considered a valid and reliable instrument to assess perceived stress.

## Introduction

Stress is experienced when an individual judges that the demand of a situation is excessive in relation to the psychological resources she or he has available to cope with it (Lazarus and Folkman, 1984). Research has highlighted that teaching is a profession with high prevalence of occupational stress (Cooper and Travers, 2012) and has identified specific potential sources of stress in this job such as workload and time pressure, organizational factors, difficult relationships with colleagues, students’ behavior (Agyapong et al., 2022; Carroll et al., 2022). Teacher stress was defined by Kyriacou (2015) as “the teacher’s experience of negative emotions such as anxiety, frustration, tension, anger, or depression, in response to the demands and pressures they face in their work as a teacher.” It is crucial to consider that stress and negative emotions in teachers can lead to psychosomatic consequences (Wettstein et al., 2021) and make some negative psychological outcomes, such as professional burnout and decreased job satisfaction, more likely to occur (Fernet et al., 2012; Salvagioni et al., 2017; Agyapong et al., 2022). Moreover, research has shown that teachers’ stress can negatively affect students’ academic achievement and quality of motivation (Shen et al., 2015; Klusmann et al., 2016; Madigan and Kim, 2021). The relevance of negative correlates of high levels of stress in teaching makes it crucial to have valid and reliable instruments to assess teachers’ stress with the aim of delivering timely and targeted interventions. This is especially true under special and unforeseen situations such as the COVID-19 pandemic, which results from recent research have clearly shown to be associated with significant reported stress among teachers (Ozamiz-Etxebarria et al., 2021). Indeed, in addition to specific and characteristic causes of stress for teachers, during the last few years the COVID-19 pandemic has added new and stressful challenges for teachers (Jakubowski and Sitko-Dominik, 2021).

There are different tools to assess stress (Crosswell and Lockwood, 2020). The Perceived Stress Scale (PSS; Cohen et al., 1983) is one of the most commonly used questionnaires for measuring perceived stress. The PSS is a self-reported questionnaire that measures the degree to which current situations are evaluated as demanding. It was developed on the basis of Lazarus’s theory of stress appraisal (Lazarus and Folkman, 1984; Lazarus, 1991). As stated by Lazarus, life circumstances are a source of stress when people appraise them as stressful, and feel they do not have the resources to cope with them. Accordingly, the PPS has the aim to evaluate the degree to which people perceive situations in their life as stressful, and their life as “uncontrollable, unpredictable, and overloaded” (Cohen et al., 1983). Specifically, the PSS assesses thoughts and feelings of an individual with respect to stressful circumstances that have arisen in the last month. The first version of the PSS consisted of 14 items; afterward, two shortened version were derived, the PSS-10 and the PSS-4 (Cohen and Williamson, 1988). The different versions of the PSS have been translated and validated in different languages, for example Spanish (Remor, 2006), Japanese (Mimura and Griffiths, 2008), Greek (Andreou et al., 2011), Chinese (Leung et al., 2010; She et al., 2021), Turkish (Kaya et al., 2019), French (Lesage et al., 2012), and Korean (Lee et al., 2015). In these studies, acceptable values of Cronbach’s alpha for the PSS-10 and PSS-14, and marginally acceptable values of Cronbach’s alpha for the PSS-4, were reported. However, the literature has shown that the psychometric properties of the 10-item version are better than those of the PSS-14 and the PSS-4, and therefore using the PSS-10 to measure perceived stress is recommended (Lee, 2012). Moreover, studies have reported a better fit of the two-factor structure compared to the one-factor model for the PSS-14 and PSS-10 (Cohen et al., 1983; Yılmaz Koğar and Koğar, 2023), while mixed findings are reported about the factor structure of the PSS-4 (Cohen and Williamson, 1988; Leung et al., 2010; Andreou et al., 2011; Mondo et al., 2021; She et al., 2021). With regard to assessment of PSS convergent validity, PPS proved to be correlated with emotional variables, such as depression and anxiety (Lee, 2012). For example, in a study aimed to evaluate the psychometric properties of a Serbian version of the PPS-10, PPS total scores were found to be positively correlated with measures of negative affect, anxiety, and depression, and moderately negatively correlated with measure of positive affect (Jovanović and Gavrilov-Jerković, 2015). Similarly, in a study aimed to validate the 14-and 10-item version of PSS in a community sample of older adults, higher levels of PSS scores were associated with lower levels of positive affect, and with higher levels of depression, anxiety, and negative affect (Ezzati et al., 2014).

This study was aimed to contribute to the validation of the Italian version of the PSS-10. The Italian translation of the PSS-10 by Andrea Fossati (2010) was used. The translation by Andrea Fossati has not been validated. To the best of our knowledge, only one study provided a validation of an Italian adaptation of the three versions of the Perceived Stress Scale (i.e., PSS-14, PSS-10, and PSS-4 items) with a sample of temporary workers (Mondo et al., 2021). In accordance with previous validation studies, Mondo et al. (2021) observed that the PSS-10 psychometric properties were better than those of the PSS-14, and the PSS-4; additionally, a better fit of the two-factor than the one-factor structure was found for all the three versions of the PSS. The differences between the 10-item PSS version by Fossati (2010) and that used in the previous Italian validation (Mondo et al., 2021) are very small and the meaning of each item is maintained. Differently from the study by Mondo et al. (2021), a sample composed of a different typology of subjects was used. Using a sample of school teachers to investigate the psychometric properties of the Italian version of the 10-item PSS represented an added value, since the previous Italian validation study considered a sample of temporary workers (Mondo et al., 2021). Specifically, in this study the Italian version of the PSS-10 was administered to a sample of pre-primary, primary, and secondary Italian school teachers during the COVID-19 pandemic.

## Aim

The aim of this study was to assess the psychometric properties, internal reliability, and convergent validity of the Italian version of PSS-10 in a sample of school teachers. According to the previous validation study of the Italian version of PSS-10, two models were compared using a Confirmatory Factor Analysis (CFA) to assess which model would provide the best fit to the data. Specifically, the study sought to assess the fit of a two-factor model of the PSS-10 consisting of two subscales, grouping positively and negatively stated items respectively, against a one-factor model consisting of a general perceived stress factor. In addition, the convergent validity of the PSS-10 was examined by considering the association with measures of subjective well-being and self-reported health. The Positive Affect and Negative Affect Schedule (PANAS; Watson et al., 1988) and a single item measuring self-reported health were used to assess correlations with perceived stress. Based on previous works, the levels of perceived stress were hypothesized to be positively correlated with negative affect and negatively correlated with positive affect. Moreover, increased stress was hypothesized to be associated with poorer self-reported health.

## Materials and methods

### Procedure

This study is part of a research that aimed at evaluating the psychological state of teachers during the COVID-19 pandemic by assessing their perceived stress and emotional experience in relation to emotion regulation and coping strategies (Messineo and Tosto, 2023). The study was a cross-sectional study, and it was conducted between March and May 2021. A sample of Italian teachers in service at pre-primary, primary and secondary schools was approached using a convenience sampling, supported by a snowball sampling technique, and invited to participate in the study, through the compilation of an online questionnaire. The link to the online questionnaire was published on the institutional website and it was sent to around 30% of the state schools available in each Italian region. Prior to accessing the online questionnaire, on the first page of the survey, teachers were informed about the aim of the research, voluntary participation, were guaranteed anonymity, and they could stop filling in the online questionnaire at any time. Participants provided informed consent to participate in the study before accessing and proceeding to answer the questionnaire.

### Ethics statement

The study was authorized by the Ethic Committee of Azienda Ospedaliera Universitaria Policlinico Paolo Giaccone of Palermo, Italy.

#### Participants

A total of 1,497 school teachers partly or wholly filled in the questionnaire. Responses from 309 participants were incomplete and thus excluded from the analysis. Furthermore, responses from other 9 participants were excluded since they reported unlikely values on questions referring to age and years of teaching. Therefore, responses from a total of 1,179 pre-primary, primary and secondary school teachers were used for the main analyses. The number of teachers that participated in the study and providing fully completed questionnaires exceeded sample size estimations based on commonly used and recognized rules of thumb (e.g., Kyriazos, 2018).

### Measures

#### Sociodemographic aspects and information about teaching career

Some questions were aimed to gather information about teachers’ sociodemographic characteristics, such as gender, age, marital status, having children. Other questions served to collect information about teachers’ careers, such as years of teaching and school level.

#### Perceived stress

The Italian translation of the 10-item Perceived Stress Scale (PSS-10; Cohen and Williamson, 1988) by Fossati (2010) was administered. Teachers were asked to rate each item indicating how often they had thought and felt a specific way during the last month on a five-point Likert scale ranging from 0 (never) to 4 (very often). The PPS-10 is composed of four positively worded items and six negatively worded items. A total PSS-10 score, ranging from 0 to 40, is computed by reversing the score of the positively stated items (items 4, 5, 7, and 8) and then summing all the item scores. Higher scores reveal a higher level of perceived psychological stress. Psychometric properties of the PSS-10 have been assessed across different countries and target populations, providing support for use of the scale as a reliable and valid measure of perceived stress (e.g., Andreou et al., 2011; Lee, 2012; Lesage et al., 2012). In the current study, the overall Cronbach’s alpha was equal to 0.88 (0.86 and 0.78 for the negative and positive subscales, respectively). McDonald’s omega for the total scores was found to be equal to 0.91; the omega coefficients for the negative and positive subscales were equal to 0.89 and 0.80, respectively.

#### Self-reported health

The following question with five response options (very good, good, fair, bad, and very bad) was administered to assess teachers’ self-reported health: “How would you evaluate your health status?” Self-reported health reflects subjective evaluation by an individual of his/her own general state of health condition (de Bruin et al., 1996). Convergent and discriminant validity, and test–retest reliability, were evaluated, providing support for use of the item as a reliable and valid measure of general health status (e.g., Lundberg and Manderbacka, 1996; Chandola and Jenkinson, 2000).

#### Positive affect and negative affect

The Italian version of the Positive Affect and Negative Affect Schedule (PANAS; Watson et al., 1988; Terracciano et al., 2003) was administered to measure teachers’ affective experience. PANAS is a 20-item questionnaire that measures positive and negative affect. Ten items measure Positive Affect (e.g., excited, and interested) and ten items measure Negative Affect (e.g., hostile, and upset). Teachers were asked to rate each item on a five-point Likert scale ranging from 1 (*very slightly or not at all*) to 5 (*extremely*) to measure the extent to which they have felt each affect during the last month. The Positive Affect score is calculated by summing the responses to the ten positive emotional items; higher scores indicate greater levels of positive affect. The Negative Affect score is computed by summing the responses to the ten items that measure negative emotions and feelings; higher scores indicate greater levels of negative affect. Each subscale’ score ranges from 10 to 50. The Italian PANAS version has proved to afford a reliable and valid measure of positive and negative affect (Terracciano et al., 2003). In the present study, Cronbach’s alphas were 0.88 and 0.87 for the Positive Affect and Negative Affect scales, respectively. Omega coefficients for the current study were found to be equal to 0.91 for the Positive Affect scale and 0.92 for the Negative Affect scale.

### Statistical analysis

All statistical analyses were carried out using R (version 4.1.2) and RStudio (version 2022.02.0). The “psych” package (version 2.1.9) was used to compute descriptive statistics and derive omega coefficient for the selected measures, and the “lavaan” package (version 0.6–10) was used to run confirmatory factor analyses. Frequency, percentages, means and standard deviations, and ranges were computed to derive participants’ characteristics in terms of socio-demographics and information about their teaching careers. Differences by gender on measures of perceived stress (PSS-10 total scores, and Positive and Negative subscales scores), Positive Affect and Negative Affect scales, and self-reported health scores were computed through a series of independent Welch’s t-tests.

A CFA of the PSS-10 was performed to compare the fit of a two-factor model against a one-factor solution of the scale. The multivariate normality assumption was assessed, and the data did not meet the assumption. As suggested by Gana and Broc (2019), the MLM estimator of the “lavaan” package was employed due to the ordinal response format (with more than 4 response categories) of the PSS-10 and violations of normality assumptions. The MLM estimator uses standard maximum likelihood to estimate the model parameters but provides robust standard errors and a Satorra-Bentler scaled test statistic (Rosseel, 2012). A CFA was conducted testing first the fit of the one-factor model (Model 1). Model 1 was specified with all 10 items loading onto one latent factor (labelled as perceived stress). The fit of a two-factor model (Model 2) was additionally tested with positively stated items (items 4, 5, 7, and 8) loading onto a first factor, labelled as Positive subscale, and negatively stated items (items 1, 2, 3, 6, 9, and 10) loading onto a second factor, labeled as Negative subscale. The goodness of fit of the models was first assessed through the Satorra-Bentler scaled chi-square; a non-significant chi-square test would suggest that the specified and observed models do not differ significantly, thus supporting the fit of the model to the data (Kline, 2011). Additional fit statistics were evaluated which included the comparative fit index (CFI), Tucker-Lewis Index (TLI), root mean square error of approximation (RMSEA), and standardized root mean square residual (SRMR). Values of CFI and TLI ≥ 0.95 (0.90–0.94), RMSEA ≤0.06 (0.07–0.08) and SRMR ≤0.08 (0.09–0.10) are considered to indicate models fitting the data very well or adequately (Hu and Bentler, 1999). In order to compare the fit of the two models, a scaled chi-square difference test was additionally computed (Rosseel et al., 2022).

In order to evaluate measurement invariance of the PSS-10 across gender, a multigroup CFA was performed testing for configural, metric, scalar, and residual invariance through four consecutive steps (Putnick and Bornstein, 2016). Configural invariance is usually run to assess the equivalence of model pattern across groups, while metric invariance addresses equivalence of factor loading. Scalar invariance is used to assess equivalence of item intercepts or thresholds, and residual or strict invariance is performed to evaluate item residuals. Configural invariance is generally tested by an evaluation of the overall fit of the model through the chi-square statistic and the additional fit statistics already proposed (i.e., CFI, TLI, RMSEA, and SRMR) (Rosseel et al., 2022). The fit of metric, scalar, and residual invariance models is usually assessed through a comparison of the fit of two nested models that are the same except for a set of restrictions in one of them. Despite a lack of consensus about the best fit indices and cutoff values for alternative fit indices under different conditions (Putnick and Bornstein, 2016), Cheung and Rensvold (2002) found that change in CFI values between comparison and nested models less than or equal to 0.01 (ΔCFI ≤0.01) indicate that the hypothesis of invariance should not be rejected. According to Chen (2007), a change in CFI of greater than or equal to − 0.01 along with a change in RMSEA of ≥0.015, or a change in SRMSR of ≥0.030 (for loading invariance) and ≥ 0.010 (for intercept invariance), are suggested as appropriate criteria which indicate a decrement in fit between models and therefore noninvariance (Chen, 2007).

The reliability of the PSS-10 was assessed by computing Cronbach’ alpha and McDonald’s omega coefficients. To conclude, bivariate correlations between PSS-10 total scores, Positive and Negative subscales scores, Positive Affect and Negative Affect scores, and measure of self-reported health were computed to assess PSS-10 convergent validity.

## Results

A sample of 1,179 teachers of pre-primary, primary, and secondary schools completed the online questionnaire. The study sample was representative of the population of Italian school teachers in terms of sex (OECD, 2023), the typology of state schools in which they were teaching, and geographical area for the North and Centre of Italy, but slightly under-representative for the South of Italy and Islands [Italian National Institute of Statistics (ISTAT), 2023]. The participants’ mean age was 47.32 years (*SD* = 10.02, range 20–67). The study sample was mainly composed by female teachers (*n* = 988, 83.8%). Overall, most participants reported they were married or living with a partner (*n* = 831, 70.5%) and had at least one child (*n* = 767, 65.1%). Moreover, participants had been working for 17 years on average, and they were teaching in pre-primary (*n* = 122, 10.4%), primary (*n* = 361, 30.6%), lower secondary (*n* = 256, 21.7%) and upper secondary schools (*n* = 440, 37.3%) at the time of filling in the questionnaire.

Table 1 reports descriptive statistics of the PSS-10, PANAS and self-reported health. The results from the Welch’s t-tests showed statistically significant differences by gender in all the measures, except for PSS-10 Positive subscale scores (see Table 1).

**Table 1:** Descriptive statistics of the PSS-10, PANAS, and self-reported health.

Variable	Gender^a^					
	Female (*n* = 988)		Male (*n* = 190)		Total (*N* = 1,179)	
	*M* (*SD*)	Range	*M* (*SD*)	Range	*M* (*SD*)	Range
PSS-10 Total score^b^	19.39 (7.04)	1–39	16.84 (6.5)	3–34	18.98 (7.01)	1–39
PSS-10 Positive subscale	7.26 (2.66)	0–15	6.78 (2.75)	0–16	7.19 (2.68)	0–16
PSS-10 Negative subscale^b^	12.12 (5.19)	0–24	10.05 (4.93)	0–24	11.79 (5.20)	0–24
PANAS Positive Affect^b^	29.79 (7.28)	11–48	30.19 (8.39)	10–50	29.85 (7.46)	10–50
PANAS Negative Affect^b^	22.67 (7.83)	10–44	20.01 (7.13)	10–50	22.24 (7.78)	10–50
Self-reported health^b^	3.87 (0.73)	1–5	4.13 (0.73)	1–5	3.91 (0.74)	1–5

M, mean; SD, standard deviation; PSS-10, 10-item Perceived Stress Scale; PANAS, Positive Affect and Negative Affect Schedule.

a Statistics by gender were derived for a total of 1,178 participants; one participant self-described as neither male nor female and was then excluded from the analyses.

b Difference by gender was statistically significant.

The *χ^2^* value for the one-factor model (Model 1), with all 10 items loading on a unique perceived stress latent factor, was 629.35 with 35 degrees of freedom (*p* < 0.001), thus suggesting that the fit of the hypothesized model to the data may not be adequate (Kline, 2011). Moreover, CFI = 0.86, TLI = 0.82, RMSEA = 0.12, and SMSR = 0.08 all indicated that the model did not fit the data adequately (see Table 2).

**Table 2:** Summary of PSS-10 factor models.

Model	Factors	Model specification	No. free parameters	*χ*^2^ (df)	CFI	TLI	RMSEA	SMSR
1	1	All items on 1 perceived stress factor	20	629.35 (35)	0.86	0.82	0.12	0.08
2	2	Positive: 4, 5, 7, 8 Negative: 1, 2, 3, 6, 9, 10	21	166.52 (34)	0.97	0.96	0.06	0.03

N = 1,179; *χ*^2^, chi square; df, degrees of freedom; CFI, comparative fit index; TLI, Tucker-Lewis index; RMSEA, root mean square error of approximation; SMSR, standardized root mean square residuals.

The unstandardized and standardized factor loadings for Model 1 are presented in Table 3. All factor loadings were statistically significant, with z statistics ranging from 11.45 to 27.63, thus showing that all the estimated parameters were significantly different from zero.

**Table 3:** Unstandardized and standardized factor loadings for the one-factor model (Model 1) and the two-factor model (Model 2).

Item	Model 1		Model 2			
			Positive Subscale		Negative Subscale	
	Unstandardized	Standardized	Unstandardized	Standardized	Unstandardized	Standardized
1. In the last month, how often have you been upset because of something that happened unexpectedly? [Nell’ultimo mese, con che frequenza si è sentito fuori di sé poiché è avvenuto qualcosa di inaspettato?]	1.00 (−)	0.69			1.00 (−)	0.70
2. In the last month, how often have you felt that you were unable to control the important things in your life? [Nell’ultimo mese, con che frequenza ha avuto la sensazione di non essere in grado di avere controllo sulle cose importanti della sua vita?]	1.12 (0.043)	0.78			1.17 (0.040)	0.78
3. In the last month, how often have you felt nervous and “stressed”? [Nell’ultimo mese, con che frequenza si è sentito nervoso o “stressato”?]	1.07 (0.041)	0.79			1.07 (0.041)	0.82
4. In the last month, how often have you felt confident about your ability to handle your personal problems? [Nell’ultimo mese, con che frequenza si è sentito fiducioso sulla sua capacità di gestire i suoi problemi personali?]	0.56 (0.039)	0.51	1.00 (−)	0.68		
5. In the last month, how often have you felt that things were going your way? [Nell’ultimo mese, con che frequenza ha avuto la sensazione che le cose andassero come diceva lei?]	0.57 (0.039)	0.51	0.98 (0.054)	0.65		
6. In the last month, how often have you found that you could not cope with all the things that you had to do? [Nell’ultimo mese, con che frequenza ha avuto la sensazione di non riuscire a star dietro a tutte le cose che doveva fare?]	0.86 (0.044)	0.62			0.86 (0.043)	0.63
7. In the last month, how often have you been able to control irritations in your life? [Nell’ultimo mese, con che frequenza ha avvertito di essere in grado di controllare ciò che la irrita nella sua vita?]	0.48 (0.042)	0.41	0.88 (0.062)	0.41		
8. In the last month, how often have you felt that you were on top of things? [Nell’ultimo mese, con che frequenza ha sentito di padroneggiare la situazione?]	0.73 (0.039)	0.64	1.30 (0.061)	0.84		
9. In the last month, how often have you been angered because of things that were outside of your control? [Nell’ultimo mese, con che frequenza è stato arrabbiato per cose che erano fuori dal suo controllo?]	0.96 (0.040)	0.68			0.96 (0.039)	0.70
10. In the last month, how often have you felt difficulties were piling up so high that you could not overcome them? [Nell’ultimo mese, con che frequenza ha avuto la sensazione che le difficoltà si stavano accumulando a un punto tale per cui non poteva superarle?]	1.25 (0.048)	0.79			1.23 (0.046)	0.79

*N* = 1,179. Dashes (--) indicate the standard error was not estimated.

Most of the items showed a substantial standardized loading on the common factor (> 0.50, as suggested by Brown, 2015), with the exception of item 7, whose loading was found to be equal to 0.41.

Model 2 was specified as a two-factor model with the four positively stated items loading onto the Positive subscale factor, and the 6 negatively stated items loading onto the Negative subscale factor. The χ^2^ value for this model was found to be equal to 166.52 with 34 degrees of freedom (*p* < 0.001) thus suggesting, as for Model 1, that the fit of the model may be problematic (Kline, 2011). On the other hand, CFI = 0.97, TLI = 0.96, RMSEA = 0.06, and SMSR = 0.03, all clearly showed that Model 2 fit the data very well (see Table 2). Table 3 shows both the unstandardized and the standardized factor loadings for Model 2. Again, all factor loadings were found to be statistically significant different from zero, with z statistics ranging from 18.02 to 28.09. All the items showed a standardized loading onto their respective factors greater than 0.50 and can thus be considered good indicators of the Positive and Negative subscale factors (Brown, 2015). Finally, the correlation between the two latent factors was equal to 0.66.

When comparing the two-factor model to the one-factor model, the significant chi-square difference statistic (Δ*χ*^2^ = 112.1, df = 1, *p* < 0.001) suggests that Model 2 provided a better fit to the data. This conclusion was also supported by the better values found for fit indexes and factor loadings of Model 2, which together suggest an overall better fit of this model to the data.

The evaluation of measurement invariance of the two-factor model of the PSS-10 was conducted on data from 1,178 participants (*n* = 988 female, 84%) given that one participant self-identified as neither male nor female and was therefore excluded from these analyses. Table 4 presents the results of the measurement invariance evaluation.

**Table 4:** Invariance models of the two-factor PSS-10 across gender.

Model	*ꭓ* ^2^	df	CFI	TLI	RMSEA	SRMR	Δ*ꭓ*^2^	ΔCFI	ΔRMSEA	ΔSRMR
Configural model	191.02***	68	0.969	0.958	0.061	0.031				
Metric model	198.80***	76	0.971	0.965	0.057	0.033	6.30	0.001	−0.004	0.002
Scalar model	209.77***	84	0.971	0.969	0.054	0.034	8.70	0	−0.003	0.003
Residual model	205.51***	94	0.973	0.974	0.050	0.035	3.33	0.002	−0.005	0.005

*N* = 1,178; *χ*^2^ = chi square; df = degrees of freedom; CFI = comparative fit index; TLI = Tucker-Lewis index; RMSEA = root mean square error of approximation; SMSR = standardized root mean square residuals. Δ refers to the difference between the parameter of comparison and nested models. ***p < 0.001.

Results from the configural invariance test showed that the model fitted the data well, as suggested by fit indices (CFI = 0.969, TLI = 0.958, RMSEA = 0.061, and SRMR = 0.031) and all factor loadings being significantly different from zero (*p* < 0.001). These results suggested that the two-factor structural patterns are not different across gender groups. The metric invariance model results showed a good fit (CFI = 0.971, TLI = 0.965, RMSEA = 0.057, SRMR = 0.033). The Δχ^2^ was not significant when compared to the configural model; this finding together with changes in fit indices generally within the suggested cut-off values provide evidence to support the metric invariance of the two-factor questionnaire. Similarly, a good fit was found for the scalar invariance model (CFI = 0.971, TLI = 0.969, RMSEA = 0.054, SRMR = 0.034) with a non-significant Δχ^2^ when the model was compared to the metric model. Finally, the residual invariance model showed a good fit as well (CFI = 0.973, TLI = 0.974, RMSEA = 0.050, SRMR = 0.035); the Δχ^2^ was found to be not significant. Overall, the last findings together suggest that the factor variances and covariances and measurement error variance for each indicator are equal across the groups. The results of measurement invariance analyses as a whole indicated that the structural factors of the PSS-10 can be considered equivalent across gender (Kline, 2011).

The values of Cronbach’s alpha and the McDonald’s omega coefficients, reported in the measures section above, revealed satisfactory reliability of the PSS-10.

Table 5 reports the Pearson’s correlation coefficients computed to assess the bivariate associations between PSS-10 (total scores, and Positive and Negative subscale scores), Positive Affect and Negative Affect scales scores, and self-reported health scores. The PSS-10 total scores and both PSS-10 subscale scores were significantly correlated with both PANAS scales and self-reported health scores, with all coefficients found to be higher than 0.40. Moreover, correlations between the PSS-10 total scores, Positive and Negative subscale scores with self-reported health were equal to −0.29, −0.25, and −0.26, respectively.

**Table 5:** Correlations for PSS-10 convergent validity.

Variable	1	2	3	4	5
1. PSS-10 total	−				
2. PSS-10 positive subscale	0.78***	−			
3. PSS-10 negative subscale	0.94***	0.53***	−		
4. PANAS positive affect	−0.52***	−0.56***	−0.41***	−	
5. PANAS negative affect	0.74***	0.48***	0.75***	−0.34***	−
6. Self-reported health	−0.29***	−0.25***	−0.26***	0.24***	−0.26***

***p < 0.001.

## Discussion

The general purpose of this study was to contribute to the validation of the Italian version of the 10-item PSS. Differently from the previous Italian validation study of the PSS-10 that used a sample of temporary workers (Mondo et al., 2021), in this study a sample composed of a different typology of subjects was used. Specifically, the present study sought to evaluate factorial structure, convergent validity, and internal reliability of the Italian translation of the 10-item PSS among a sample of Italian pre-primary, primary, and secondary school teachers.

First, a CFA was performed to assess factorial validity and define the best fitting model of the scale. The results of the CFA showed that the two-factor structure of the Italian version of the PSS-10 provided a better fit to the data than the one-factor model. This finding is consistent with the validation study of the Italian version of the PSS with a sample of temporary workers (Mondo et al., 2021), in which the authors found that the two-factor model for the PSS-10 showed a better fit than the single-factor model. In addition, this result is in line with other previous studies carried out in other contexts and with diverse typologies of populations, both in clinical (e.g., Leung et al., 2010; Lee et al., 2015; Soria-Reyes et al., 2023) and nonclinical setting (e.g., Kaya et al., 2019; She et al., 2021). Recently, a systematic review by Yılmaz Koğar and Koğar (2023), aimed to examine the factor structure of PSS, showed that the model that best explained the factor structure of the PSS-10 was the two-factor model.

A measurement invariance analysis was performed to evaluate the measurement invariance of the PSS-10 across gender. The results from this analysis showed that the two-factor model of the PSS-10 can be considered equivalent for male and female teachers, indicating measurement invariance across gender. This finding is in accordance with previous studies carried out with different typology of subjects, such as adolescents (e.g., Liu et al., 2020; Chen et al., 2024), and university students (Denovan et al., 2019; Lee, 2023).

Concerning the internal consistency of the 10-item PSS and its two factors, Cronbach’s alpha and McDonald’s omega coefficients indicated satisfactory reliability. This result is consistent with the previous study examining the psychometric properties of the Italian version of the PSS-10 (Mondo et al., 2021) and with other validation studies of the 10-item PSS, in which acceptable values of Cronbach’s alpha and McDonald’s omega coefficients were reported (e.g., Lee, 2012; She et al., 2021; Soria-Reyes et al., 2023).

Evidence of validity was assessed through the evaluation of the correlation of the PSS-10 scores with measures of emotional well-being and self-rated health. As hypothesized, the convergent validity of the scale was supported by observation of the positive correlation of the PSS-10 scores with Negative Affect, and of the negative correlation with Positive Affect of PANAS. This result is in line with previous studies. For example, in a study aimed to evaluate the psychometric properties of a Serbian version of the PPS-10, the PPS total scores were found to be positively correlated with measure of negative affect, and moderately negatively correlated with measure of positive affect (Jovanović and Gavrilov-Jerković, 2015). Similarly, in a study carried out by Ezzati et al. (2014), it was that found that higher levels of total PSS scores were associated with higher levels of negative affect, and lower levels of positive affect. As hypothesized, the PSS-10 also showed good convergent validity with a measure of self-reported health. Indeed, as expected in accordance with the findings by Cohen and Williamson (1988), the higher stress scores as measured by the PSS-10 were associated with poorer self-reported health. These findings are consistent with other studies. For example, in a study by Leung et al. (2010), carried out with a sample of Chinese cardiac sufferers with a smoking habit, the authors found that the total scores of PSS correlated negatively with poorer perceived health status in the last three months. A similar finding was observed by Schneider et al. (2020) in a study performed to test a German adaptation of the PSS-10 among clinical and nonclinical subjects. Specifically, the authors observed a negative and significative correlation between the total PSS scores and the WHO-Five Well-Being Scale.

In this study, the relationship between perceived stress and gender was examined. In this context, females showed higher PSS scores than males. As regards this relationship, the existing literature presents contrasting results. For instance, some studies showed that females reported higher PSS scores than males (Remor, 2006; Leung et al., 2010; Andreou et al., 2011; Lesage et al., 2012; Ezzati et al., 2014), while according to other studies there was no statistical difference between PSS scores of males and females (Cohen et al., 1983; Mondo et al., 2021). Overall, these findings suggest that further investigations are required to clarify this issue.

In summary, the findings of this study offer further support for the good psychometric properties and convergent validity of the 10-item PSS in the Italian context.

### Limitations

This study has some limitations. First, a limitation is due to the fact that the PSS is a self-reporting instrument; this may have led to a response bias. Therefore, future research should also use different typologies of measures. Moreover, the inclusion of teachers only in this study may limits the generalizability of the findings of this study to a wider population.

## Conclusion

The findings from this study show that the Italian version of the 10-item PSS has good psychometric properties and satisfactory reliability. Its convergent validity was supported by correlation analyses with measures of emotional well-being and self-rated health. Overall, the Italian version of the PSS-10 can be considered a valid survey to measure perceived stress.

## Data availability statement

The raw data supporting the conclusions of this article will be made available by the authors, without undue reservation.

## Ethics statement

The studies involving humans were approved by Ethic Committee of Azienda Ospedaliera Universitaria Policlinico Paolo Giaccone of Palermo, Italy. The studies were conducted in accordance with the local legislation and institutional requirements. The participants provided their written informed consent to participate in this study.

## Author contributions

LM: Conceptualization, Data curation, Investigation, Methodology, Supervision, Writing – original draft, Writing – review & editing. CT: Data curation, Formal analysis, Writing – original draft, Writing – review & editing.
